# Prevalence, incidence of and risk factors for vertebral fracture in the community: the Vietnam Osteoporosis Study

**DOI:** 10.1038/s41598-023-50145-w

**Published:** 2024-01-02

**Authors:** Hoa T. Nguyen, Bao T. Nguyen, Thi H. Nhung Thai, An V. Tran, Tan T. Nguyen, Tam Vo, Linh D. Mai, Thach S. Tran, Tuan V. Nguyen, Lan T. Ho-Pham

**Affiliations:** 1 Department of Internal Medicine, University of Medicine and Pharmacy, Hue University, Hue, Vietnam; 2 Can Tho University of Medicine and Pharmacy, Faculty of Medicine, Can Tho, Vietnam; 3 Biomedicine Research Center Pham Ngoc Thach University of Medicine, Ho Chi Minh City, Vietnam; 4 Saigon Precision Medicine Research Center, Ho Chi Minh City, 70000 Vietnam; 5 Bone and Muscle Research Group Ton Duc Thang University, Ho Chi Minh City, Vietnam; 6 Tam Anh Research Institute, Tam Anh Hospital, Ho Chi Minh City, Vietnam; 7 School of Biomedical Engineering, University of Technology Sydney, Ultimo, Australia

**Keywords:** Osteoporosis, Rheumatology, Risk factors

## Abstract

The epidemiology of vertebral fractures (VF) in underrepresented populations is not well-documented. This cohort study was part of a longitudinal osteoporosis research project with the aim of determining the prevalence, incidence, and risk factors for VF. 401 individuals (155 men) aged 50 years and older without a clinical diagnosis of VF were took radiographs at baseline and 2 years later. VF were ascertained using the Genant's semi-quantitative method. Bone mineral density (BMD) of femoral neck and lumbar spine were measured by dual-energy X-ray absorptiometry (Hologic Inc). The association between VF and risk factors was analyzed by the multiple logistic regression. The 95% confidence interval for prevalence and incidence was estimated by exact Poisson test. At baseline, the prevalence of VF was 12.2% (n = 49, 95% CI 9.0–16.2%) and increased with advancing age with one-fifth of those aged 70 and older having a VF. During the follow-up period, we observed 6 new VF, making the incidence of 6.6/1000 person-years (n = 6, 95% CI 2.4–14.3). The risk of prevalent VF was associated with male gender (OR: 2.67; 95% CI 1.28–5.87) and T-score at the femoral neck (OR per one SD decrease: 1.1; 1.03–1.17). These data indicate that VF is common among adults, and that lower femoral neck BMD was a risk factor for VF.

## Introduction

Vertebral fracture due to osteoporosis can be an important health problem because it is associated with serious consequences. Patients with vertebral fracture are more likely to experience pain and disability than those without a vertebral fracture^1^. As a result, vertebral fracture is also associated with reduced quality of life^2^. More importantly, a vertebral fracture is also associated with an increased risk of mortality, such that women with a vertebral fracture having 1.2-fold age-adjusted increase risk of mortality^3^, and the risk was substantially increased among those with a vertebral deformity^4^. Moreover, patients with a vertebral fracture are at greater risk of subsequent vertebral fracture^5^. Collectively, these studies suggest that severe vertebral fracture is a significant clinical problem among elderly people.

Vertebral fracture is considered the hallmark of osteoporosis because it is the most common form of osteoporotic fractures^6–8^. In Western populations, vertebral fracture affects up to 30% of men and women aged 50 years and older^9–11^. In Asian populations 50+ years, the prevalence of vertebral fractures ranged between 12 and 25%^12,13^. However, the incidence of vertebral fractures has not been well documented due to lack of longitudinal study. The EPOS study in Europe showed an overall incidence of 10.7/1000 person-years in women and 5.7/1000 person-years in men^14^. However, the actual incidence is likely greater because a large number of fractures do not come to clinical attention^15^ and up to three-quarters to two-thirds are clinically undiagnosed^16,17^. The burden of vertebral fracture is expected to increase in the future as the global population is aging.

Although the epidemiology of vertebral fractures has been well studied in Caucasian populations, it remains under-documented in underrepresented populations. Most of these populations have recently experienced rapid urbanization and significant changes in lifestyle that adversely affect population health. In order to contribute to the gap of literature, the present study sought to determine the prevalence, incidence of and risk factors for vertebral fractures in men and women over 50 years in a Vietnamese population. We consider that the population-based data reported here contribute to the international literature on osteoporosis and serve as fundamental background to guide national healthcare strategies.

## Results

### Prevalence of vertebral fractures

The study included 401 individuals (155 men) whose mean age was 59 years, with no significant difference in age between men and women. There was no significant difference in body mass index and lumbar spine BMD between genders. As expected, men were more likely to be current smokers than women (40.6% and 0.4%, P < 0.001). On average, men had a greater femoral neck BMD than women (p = 0.001) (Table 1).

**Table 1:** Characteristics of 401 participants stratified by gender.

Factors	Men (n = 155)	Women (n = 246)	P-value
Age (years), mean (SD)	58.8 (5.9)	59.7 (6.5)	0.151
Body mass index (kg/m^2^), mean (SD)	23.0 (3.1)	23.4 (3.0)	0.229
Current smoking (yes), n (%)	63 (40.6)	1 (0.4)	< 0.001*
History of fall (yes), n (%)	15 (9.7)	37 (15.0)	0.160
History of fracture (yes), n (%)	6 (3.9)	12 (4.9)	0.821
T-score at the femoral neck, median (IQR)	− 0.98 (− 1.57, − 0.43)	− 1.48 (− 2.12, − 0.86)	0.001*
T-score at lumbar spine, median (IQR)	− 1.16 (− 1.67, − 1.14)	− 1.24 (− 1.94, − 0.52)	0.086

*IQR* interquartile range, *SD* standard deviation.

*Significant.

Using the Genant's criteria, we found 49 of the 401 individuals had a vertebral fracture, yielding the prevalence of 12.2% (95% CI 9% to 16.2%). Men had a higher prevalence of vertebral fractures than women (14.8% vs 10.6%). In either sex, the prevalence was increased with advancing age, from 10.8% among those aged 50–59 to 19% among those aged 70 and older. Wedge fractures was the most common type of fractures (89.3%). In addition, most fractures (80.3%) were grade 1 (Table 2).

**Table 2:** Prevalence (%) of vertebral fractures among 401 individuals classified by age, gender, and type of fracture.

Factors	N/total	Prevalence (95% CI)
Any vertebral fracture	49/401	12.2% (9.0, 16.2)
By gender		
Men	23/155	14.8% (9.4, 22.3)
Women	26/246	10.6% (6.9, 15.5)
By age group		
50–59	26/240	10.8% (7.0, 15.9)
60–69	19/140	13.5% (8.2, 21.2)
70+	4/21	19.0% (5.2, 48.8)
Type of fracture		
Wedge	50/56	89.3% (66.2, 117.7)
Biconcavity	1/56	1.7% (0.04, 9.9)
Compression	5/56	8.9% (2.9, 20.8)
Grade of fracture		
1	45/56	80.3% (58.6, 107.5)
2	8/56	14.3% (6.2, 28.1)
3	3/56	5.4% (1.1, 15.7)

**Figure 1:**
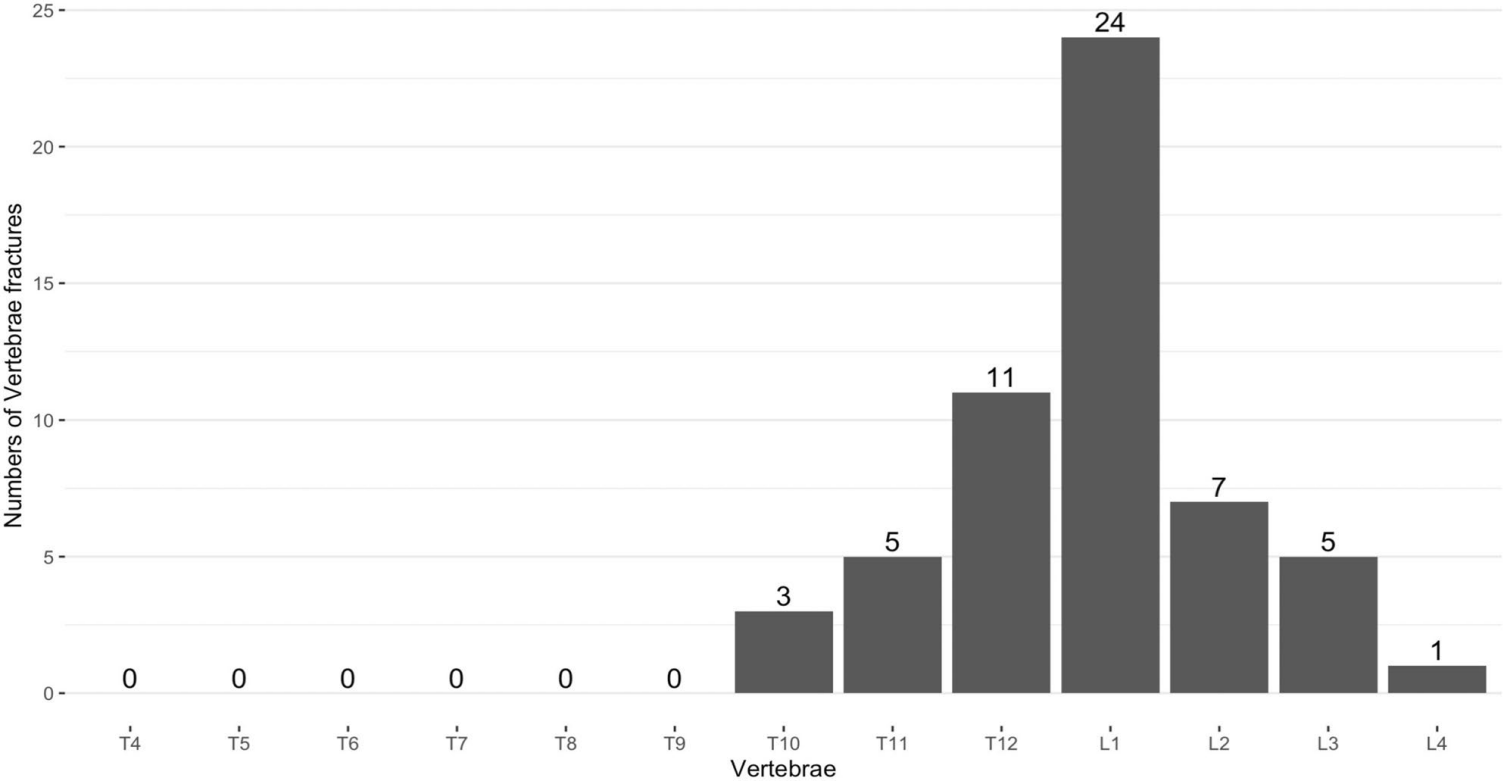
Distribution of vertebral fractures from T4 to L4.

Based on the analysis of each individual spinal column, T12 and L1 were the most affected vertebrae (Fig. 1). More than one-thirds of all fractured cases occurred at L1 vertebrae.

### Risk factors of vertebral fractures

Univariate analysis revealed the following risk factors for vertebral fractures: male gender, advancing age, greater body mass index, low bone mineral density, fracture history, fall history, and current smoking. However, in multivariable analysis, only male gender (OR: 2.67; 95% CI 1.28–5.87), and lower femoral neck BMD T-score (OR per SD decrease: 1.1, 1.03–1.17) were independently associated with the risk of prevalent vertebral fracture (Table 3).

**Table 3:** Risk factors of radiographic vertebral fractures: the result of logistic regression analysis.

Factor	Unit of comparison	Odds ratio (95% CI)
Gender	Men	2.67 (1.28–5.87)
Age (years)	+ 5	0.95 (0.74–1.23)
Body mass index (kg/m^2^)	+ 1	1.05 (0.94–1.17)
T-score at the femoral neck	− 1	1.1 (1.03–1.17)
T-score at the lumbar spine	− 1	0.98 (0.94–1.02)
History of fracture	Yes	1.91 (0.55–6.65)
History of fall	Yes	1.75 (0.77–3.98)
Current smoking	Yes	0.64 (0.25–1.65)

During the 2-year follow-up we observed 6 new vertebral fractures, making the overall incidence of vertebral fracture of 6.6 per 1000 person-years (95% CI 2.4 to 14.3 per 1000 person-years).

## Discussion

Vertebral fracture is the hallmark of osteoporosis, but most vertebral fractures do not come to clinical attention. The present study which focused on a population of adults that is underrepresented in osteoporosis research, revealed that approximately 12% of individuals have a vertebral fracture. Additionally, the study showed that the incidence of new fractures was approximately 1% per year. We further found that the risk of fracture was associated with male gender and lower femoral neck bone mineral density. Our study is one of the very few investigations into vertebral fractures in Vietnam, and our original data make a valuable contribution to the global literature on osteoporosis, particularly in underrepresented populations.

The prevalence of vertebral fractures found in our study is in line with what has been reported in other populations around the world. For example, a study of 19 European countries showed that the prevalence of asymptomatic vertebral fractures among people aged 50 to 79 was between 6 and 21%^11^. The Latin America Vertebral Osteoporosis Study (LAVOS) found that 11,18% of people had vertebral fractures^9^. In Asia, the China Osteoporosis Prevalence Study^18^ estimated that the prevalence of vertebral fracture was ~ 10% in men and women, and this estimate is highly consistent with our estimate. A study in Taiwan using radio morphometric methods found that 13% of men and 20% of women aged 65 and older had vertebral fractures^19^. Although our estimate of prevalence is lower than a previous study of postmenopausal women of Vietnamese background using the same Genant’s assessment method^20^, our estimate is still consistent with previous estimates in both Caucasian and Asian populations.

Our data suggest that wedge deformity accounted for the majority (89%) of all vertebral fractures, confirming observations in Netherlands and India, where wedge deformity was also a major vertebral fracture among adults^21,22^. We also note that the majority of vertebral fractures are grade 1 (80%), and this is also comparable with previous studies^21,23–25^.

While the prevalence of vertebral fractures in Caucasian populations has been extensively studied, the incidence of these fractures has not received as much attention. Our study found that the incidence of new asymptomatic vertebral fractures was approximately 1% over a two-year period. This estimate was based on a small number of events, but it is slightly lower than that of Korea^26^, Switzerland^27^, and Netherlands^28^. Notably, studies in Caucasian populations tended to produce lower estimates than those in Asian populations. For example, a study in the 1990s in the United States found that the incidence rate for symptomatic vertebral fractures in white women was 0.1 per 100 person-years^16^, higher than a major nationwide study in the UK which reported an incidence of 0.032 per 100 person-years in men and 0.06 per 100 person-years in women aged 20 and above^29^. However, these estimates are likely underestimation because most vertebral fractures are asymptomatic and did not come to clinical attention.

We found that lower femoral neck BMD, not lumbar spine BMD, was associated with an increased risk of vertebral fracture, confirming previous findings^30,31^. This finding is consistent with that of the previous authors which an 0.1–0.12 g/cm^2^ lower femoral BMD was associated with a 1.5–1.8-fold increase in vertebral fracture risk^32,33^. The lack of association between spinal BMD and vertebral fracture in our study is probably due to the poor predictive value at this bone density site that has been well observed in many studies. Our findings, together with previous findings, support the International Society for Clinical Densitometry recommendation that a vertebral fracture assessment test should be indicated for those with osteopenia at the femoral neck.

Our findings have important public health implications for the prevention of osteoporosis in the general community. Our data provide evidence for using BMD to identify individuals at high risk of vertebral fractures for preventive measures. In addition, this study also provides data on the prevalence and incidence of vertebral fractures in a population that is often underrepresented in the literature of osteoporosis. In Vietnam, osteoporosis has not received adequate attention due to 'competing diseases' such as infection, cancer, and type 2 diabetes. However, our finding suggests that osteoporosis should be considered a national health priority in Vietnam.

Our findings should be interpreted within the context of strengths and potential weaknesses. This study was based on a well-characterized cohort with the participants drawn from the general population by a standardized sampling scheme, ensuring representativeness and external validity. We controlled the internal validity of the data by three independent readers of radiographs. However, due to the relatively small sample size, it was not possible to analyze the factors associated with the incidence of vertebral fractures. Moreover, the participants in this study were drawn from a major city, and the findings may not be generalizable to rural populations.

In summary, this cross-sectional and prospective study was conducted on individuals aged 50 years and older in Vietnam revealing a relatively high prevalence of vertebral fractures (12%), with an annual incidence of new fractures of approximately 1%. The prevalence and incidence are comparable to that found in previous studies of Caucasian populations. Our finding of the association between low bone density and fracture risk implies that public health efforts should prioritize the use of femoral neck bone mineral density to identify high-risk men and women for vertebral fractures.

## Methods

### Study design and participants

The present study was part of the Vietnam Osteoporosis Study (VOS) in which study procedures and protocols have been published previously^34^. Briefly, participants were recruited from the general community in Ho Chi Minh City and many other regions. Various outreach methods, such as media campaigns and community meetings (e.g., in churches, temples, and senior citizen groups), were employed to engage participants. We distributed the Vietnamese-language flyers described the study's objectives, procedures, benefits, and potential risks. Participants did not receive any monetary incentives but were provided with a complimentary health check-up and lipid analyses. The data was collected in 2 years. Study subjects visited the Bone and Muscle Research group located at Ton Duc Thang University and managed by Saigon Precision Medicine Research Center (SAIGONMEC) for clinical assessment and evaluation. All individuals voluntarily took part in the study, and no financial incentives were offered. The Study's procedure and protocol were approved by the research and ethics committee of the People's Hospital 115 (Approval No. 47/BV-NCKH dated July 30, 2015). The study was conducted according to the ethical principles of the Declaration of Helsinki, and all participants gave written informed consent.

Sample size determination was based on the data from a previous study in the Chinese population which found that the prevalence of vertebral fractures was ~ 10%^18^. Using this prevalence as an initial estimate, and with a marginal error of 3%, we estimated that a sample size of 384 individuals was required for the present study.

**Figure 2:**
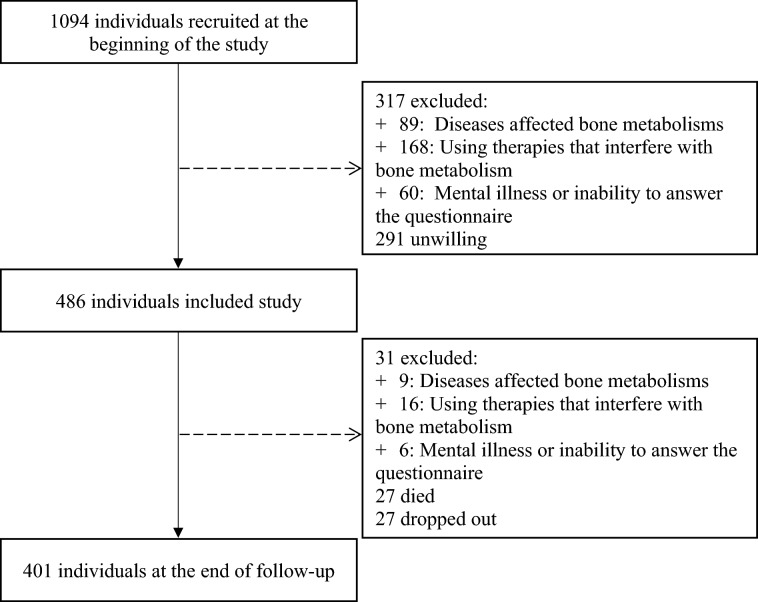
Flowchart of the recruitment of participants.

The inclusion criteria for this study were: all individuals aged 50 years and older and agreed to participate in the study. Participants were excluded from the study if they had any disease that affected bone metabolisms, such as renal failure, hypothyroidism, diabetes mellitus, malabsorption syndrome, or bone cancer. We also excluded participants who had previous or current use of therapies that interfere with bone metabolism (e.g., corticosteroid, antidiabetic medications, heparin, warfarin, thyroxin, and estrogen), mental illness or inability to answer the questionnaire. Figure 2 shows the disposition of the analyzed patients.

### Data collection

A standardized questionnaire was used to collect demographic and clinical data. The questionnaire was divided into 9 sections^34^: anthropometric and demographic characteristics; lifestyle factors; nutrition and dietary calcium intakes; reproductive history (women); clinical (eg diseases) history; medication history; fracture history; fall history; and biochemical test results. The general characteristics included age, smoking, history of fracture, history of fall, weight, height. Body weight was measured using an electronic balance with indoor clothing without shoes. Height was measured on a handheld stadiometer without shoes. Body mass index (BMI) was derived as the weight in kilograms divided by the square of the height in meters.

### Assessment of vertebral fracture

Standard lateral and anterior–posterior lumbar spine radiographs (FCR Capsula XLII Fujifilm Corp, Tokyo, Japan) were taken with a 101.6 cm tube-to-film distance and were centered at L2. Radiographic fracture (referred as vertebral fracture in this study) was evaluated by the Genant’s semi-quantitative (SQ) method^35^. The first author and the second author independently read the same radiographs. When there is a discrepancy between the two original authors' X-ray readings, the corresponding author evaluates their results and rereads the radiographs. The final X-ray results are decided by the corresponding author. Any discrepancies were solved by a joint consensual reading. The kappa coefficient among readers was 0.67.

Vertebral fractures were classified using Genant's semi-quantitative method, which includes three types: wedge, concavity, and compression. The severity of vertebral fractures (grade 1, grade 2, or grade 3) was assessed based on Genant's semi-quantitative criteria, where the reduction in vertebral anterior, middle, and/or posterior height was categorized as 20–25% (grade 1), 25–40% (grade 2), and over 40% (grade 3)^35^.

### Bone mineral density measurement

Bone mineral density (BMD) was measured using the Hologic Horizon densitometer (Hologic Corp., Bedford, MA, USA) at the femoral neck (FN) and lumbar spine (LS). Before every measurement, the densitometer was calibrated using a standard phantom. The precision error (%CV) was 1.8% for the femoral neck BMD and 2% for the lumbar spine BMD.

T-score was converted from BMD as the difference between an individual's BMD (${BMD}_{i}$) and the population mean (mean) taken as aged between 20 and 30 years, and then standardized by the standard deviation (SD): T-score = BMD_i_ – mean)/SD; where 'mean' and 'SD' were derived from the local population that has been published previously^36^. According to the World Health Organization (WHO), an individual with T-score ≤ − 2.5 is considered to have osteoporosis.

### Data analysis

The prevalence of vertebral fractures was estimated as the ratio of the number of vertebral fractures over the population at risk. The incidence of vertebral fractures was determined by the number of new fractures appearing in the second radiograph. We used the exact Poisson test to estimate the 95% confidence interval (95% CI) for the population prevalence and incidence. This computation was implemented in the R Statistical Environment^37^. We used a multivariable logistic regression model to identify the risk factors for vertebral fractures. A P-value of less than 0.05 was considered statistically significant. The magnitude of association between a risk factor and vertebral fracture was expressed in odds ratio and 95% CI. We considered the following potential risk factors: age, current smoking, body mass index history of fracture, history of fall, T-score at the femoral neck and T-score at the lumbar spine.

### Ethics approval

The study was approved by the Ethics Committee of the People’s Hospital 115. The study was conducted according to the ethical principles of the Declaration of Helsinki.

### Informed consent

All participants gave written informed consent.

## Data Availability

The datasets generated during and/or analysed during the current study are available from the corresponding author on reasonable request.
